# Risk factors for inadequate antibody response to primary rabies vaccination in dogs under one year of age

**DOI:** 10.1371/journal.pntd.0005761

**Published:** 2017-07-31

**Authors:** Ryan M. Wallace, Anna Pees, Jesse B. Blanton, Susan M. Moore

**Affiliations:** 1 Poxvirus and Rabies Branch, Centers for Disease Control and Prevention, Atlanta, Georgia, United States of America; 2 Rabies Laboratory, Kansas State Veterinary Diagnostic Laboratory, Kansas State University Rabies Laboratory, Manhattan, Kansas, United States of America; Wistar Institute, UNITED STATES

## Abstract

Ensuring the adequacy of response to rabies vaccination in dogs is important, particularly in the context of pet travel. Few studies have examined the factors associated with dogs’ failure to achieve an adequate antibody titer after vaccination (0.5 IU/ml). This study evaluated rabies antibody titers in dogs after primary vaccination. Dogs under one year of age whose serum was submitted to a reference laboratory for routine diagnostics, and which had no prior documented history of vaccination were enrolled (n = 8,011). Geometric mean titers (GMT) were calculated and univariate analysis was performed to assess factors associated with failure to achieve 0.5 IU/mL. Dogs vaccinated at >16 weeks of age had a significantly higher GMT compared to dogs vaccinated at a younger age (1.64 IU/ml, 1.57–1.72, ANOVA p < 0.01). There was no statistical difference in GMT between dogs vaccinated <12 weeks and dogs vaccinated 12–16 weeks (1.22 IU/ml and 1.21 IU/ml). The majority of dogs failed to reach an adequate titer within the first 3 days of primary vaccination; failure rates were also high if the interval from vaccination to titer check was greater than 90 days. Over 90% of dogs that failed primary vaccination were able to achieve adequate titers after booster vaccination. The ideal timing for blood draw is 8–30 days after primary vaccination. In the event of a failure, most dogs will achieve an adequate serologic response upon a repeat titer (in the absence of booster vaccination). Booster vaccination after failure provided the highest probability of an acceptable titer.

## Author summary

For rabies prevention and control it is important that pets and domestic animals be vaccinated, this is especially relevant for rabies-free areas of the world which tightly control dog and cat entry to ensure rabies is not imported. Adequate response to rabies must be proven by testing the pet’s serum for rabies antibodies. Failure to produce an adequate antibody response is related to various factors such as pet health, vaccine type, timing of vaccination and subsequent blood sampling as well as age. Identifying the ideal vaccination and blood drawing schedules. Unique factors, such as maternal antibody interference, affect the ability of young dogs to mount a robust immune response to vaccination; this group of pets has not previously been studied. This study looked at rabies antibody result from dogs under one year of age after a single vaccination to determine the timing of vaccination and a blood sampling that ideally would ensure an antibody response sufficient to prove the vaccination was effective for rabies protection.

## Introduction

Recommendations for the vaccination of dogs against rabies differs based upon the endemic rabies status in a country or region. Countries that have enzootic circulation of rabies in dogs are encouraged to vaccinate a minimum of 70% of the dog population each year, for 5–7 years, to achieve elimination [1]. For these countries, all dogs should be vaccinated when campaigns are held, regardless of age or previous vaccination status. Once the canine rabies virus variant has been eliminated, vaccination recommendations often vary depending on the transmission patterns of wildlife rabies in the region. In settings where wildlife rabies reservoirs pose a threat to domestic animals, dog vaccination may still be important from a public health perspective, but the 70% threshold for elimination purposes is no longer applicable [2]. In the United States, a canine rabies-free country, most rabies vaccines are licensed for dogs older than 12 weeks of age, and the National Association for State Public Health Veterinarians recommends a booster vaccination one year later. Frequency of re-vaccination is often determined by local policy (either annual or tri-annual booster vaccination).

Re-incursion of the canine rabies virus may pose negative public health and fiduciary consequences. Therefore, countries that have eliminated this variant often require proof of vaccination and/or proof of adequate rabies antibody titer for dogs entering the country. For example, dogs entering New Zealand, a rabies-free country, are required to have received a rabies vaccine given no less than six months prior and not more than one year prior to the date of entry [2,3]. In addition, proof of a rabies antibody titer of at least 0.5 IU/ml from a blood sample collected not less than three months and not more than 24 months prior to entry is required. Depending on the jurisdiction, dogs without proof of vaccination may be turned away, euthanized, or granted access in extenuating circumstances through government monitoring programs [4].

Rabies vaccination failures in dogs, although rare, have been documented and falsification of records has resulted in the entry of canine rabies virus variant infected dogs into rabies-free countries [5,6]. Therefore, in addition to veterinary records, many countries require that dogs seeking entry into rabies-free jurisdictions also provide serologic evidence of vaccination. A rabies neutralizing antibody titer ≥ 0.5 IU/ml is defined by WHO and OIE as the minimum post-vaccination antibody level [1,7]. However, several studies have reported that roughly 10% of rabies vaccine naïve dogs fail to reach this threshold after primary vaccination [8,9]. The immune response to primary vaccination may be affected by intrinsic factors such as genetics, as well as extrinsic factors such as long delays in the time from vaccination to antibody titer measurement [10–12]. Vaccination costs (including veterinary fees) average $65.00 USD, and the typical cost to verify an immunologic response is $150.00 USD (Kansas State University). Therefore, failure to detect an adequate antibody titer could result in costs of over $400.00 (2 rabies vaccinations and 2 titer draws) per failed titer check. Identifying predisposing factors and describing the correct timing for primary vaccination, booster vaccination, and blood draws for titer verification can have both a health impact for the dog as well as an economic impact for the owner.

This study evaluates the factors associated with failure to reach adequate titers among dogs that are under one year of age and had no history of vaccination. This cohort has not previously been studied for risk factors and specifically for blood sampling delay. Knowledge of the ideal timing of rabies vaccination and blood draw would help veterinarians prepare pets for export to rabies-free areas as well as provide more information that guides optimal protection from rabies.

## Methods

Kansas State University (KSU) Rabies Laboratory serves as a national reference laboratory for confirming rabies neutralizing antibody levels in domestic animals and humans. KSU performs rabies serology for international pet travel, clinical trial studies of rabies biologics for global use, and research of oral rabies vaccine response in wildlife as well as other rabies research. Serum samples and accompanying dog demographic data were submitted from 162,739 animals to the KSU Rabies Laboratory between 2006 and 2010 for the detection of rabies antibody titer levels prior to pet travel. Samples are tested via Fluorescent Antibody Virus Neutralization (FAVN) testing and data from submission forms are entered into Microsoft Access and the Universal Veterinary Information System. Submission forms include the name and address of the submitting clinic or laboratory and animal information (species, name, identification number, birthdate, sex, breed, vaccination history, and serum draw date).

This study was conducted to evaluate the detection of rabies antibody in rabies vaccine naïve dogs after primary vaccination. Therefore, the dataset was limited to dogs under one year of age (n = 13,061) and was further restricted to those with no prior documented history of rabies vaccination (n = 8,011). Submission forms without an identification number, birth date, serum draw date, and vaccination history were omitted from the study.

The FAVN assay is performed following the published OIE procedure [13]. Many countries and international organizations consider an acceptable titer to be 0.5 IU/mL [14]. For purposes of pet travel, three-fold serum dilutions are carried out to 1:81 to allow discrimination between samples less than 0.5 IU/mL and those greater than or equal to 0.5 IU/mL. Dogs for which titer results were not carried out to end-point determination (i.e., above the upper limit of detection of the assay), a weighted average among all dogs in the reported titer group, and the next two higher dilutions, was calculated. This weighted titer was used as a proxy for the end-point titer for purposes of data analysis. Dogs with sera titers less than 0.5 IU/ml were considered to have a failed test and a titer greater than or equal to 0.5 IU/ml was considered to have a passing test [1,14]. Canine samples were further categorized into the following cut-off titer groups: <0.5 IU/ml (Low responders), ≥0.5—<1.5 IU/ml (Moderate responders), 1.5—>7.9 IU/m (High responders). Unless otherwise noted, only the initial FAVN titer after primary vaccination was analyzed. Repeated titers for dogs that failed to reach a titer of 0.5 IU/ml, for which additional data were available, were analyzed separately.

Dogs were classified into three groups based on their age at primary vaccination: less than 12 weeks of age, 12–16 weeks of age (on-time), and greater than 16 weeks of age. Most rabies vaccines in the US market are licensed for dogs older than 12 weeks of age, therefore dogs with primary vaccination earlier than 12 weeks were considered early vaccinations [2]. Most state regulations require that a dog be vaccinated prior to 16 weeks of age. Therefore, dogs receiving rabies vaccination later than 16 weeks were considered late immunizations.

The time period between initial vaccination and sample draw was calculated based on dates provided on submission forms and defined as the *draw interval*. Eight draw intervals categories were defined: 1) ≤ 3 days, 2) 4 days to ≤ 7 days, 3) 8 days to 14 days, 4) 15 days to 30 days, 5) 31 days to 90 days, 6) 91 days to 180 days, 7) 180 days to 270 days, and 8) > 270 days.

The submission form accompanying the sample included an entry for dog breed. Dogs with only one dog breed listed were categorized as “Pure Breed” and dogs with multiple breeds listed or specifically noted as “Mix” on submission forms were considered “Mixed Breed”. The analysis did not delineate between different breeds but rather assessed the variables of pure breed versus mixed breed. Breed was also used to categorize dogs into 5 size groups: toy, small, medium, large, and giant. The American Kennel Club (AKC) has defined each size group with a weight range and the breeds included in each size group. The AKC groupings were used to define the size groups in this study.

### Statistical analysis

**Fig 1:**
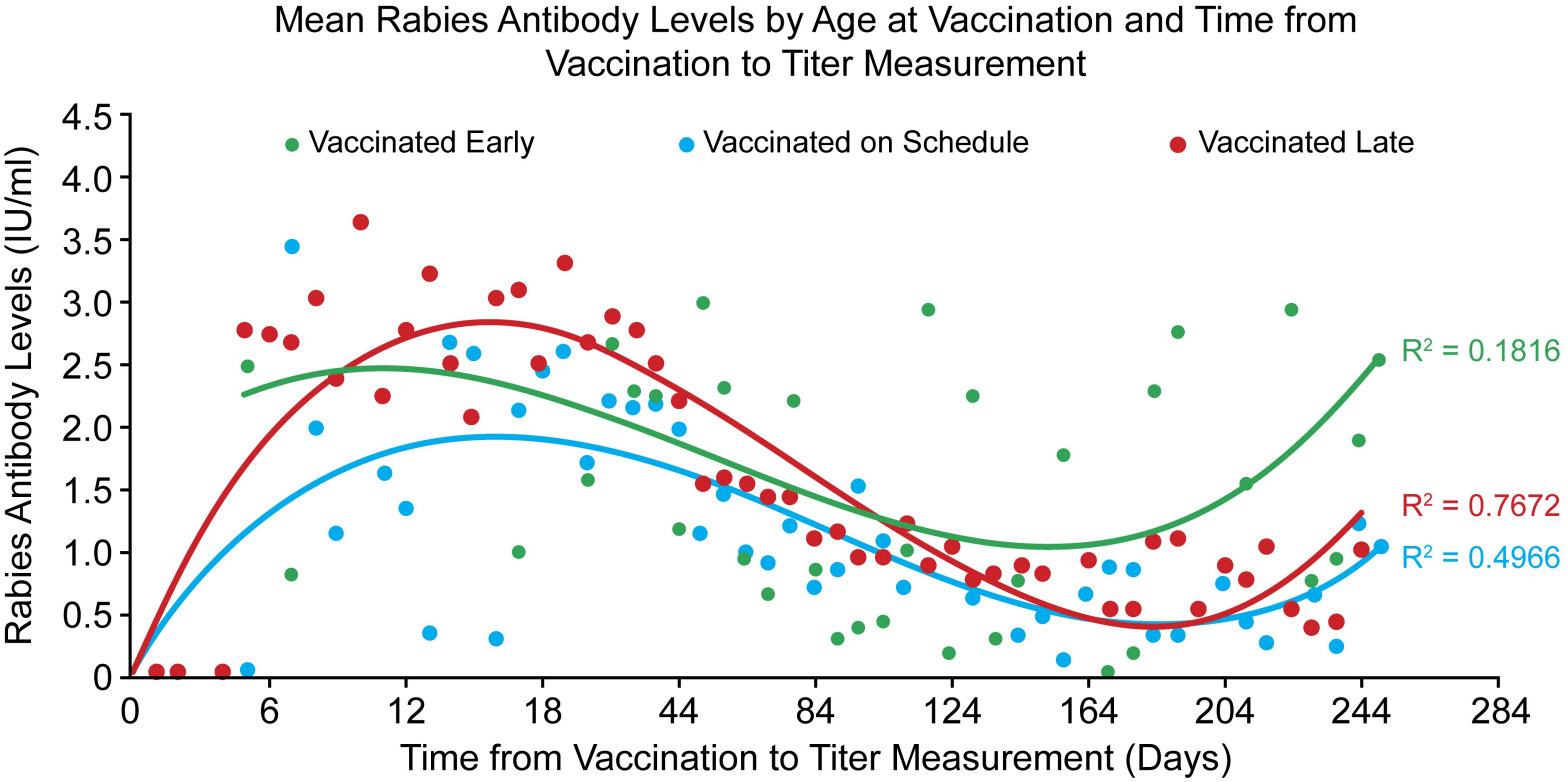
Rabies antibody levels after primary vaccination per vaccination age (early, on schedule, late) and day of draw. *Data truncated at 250 days due to small sample size after this draw interval*. *Vaccinated Early*: *y = 0*.*0001x*^*3*^*–0*.*0091x*^*2*^ *+ 0*.*1602x + 1*.*6497*. *Vaccinated on Schedule*: *y = 0*.*0002x*^*3*^*–0*.*0179x*^*2*^ *+ 0*.*4117x + 0*.*019*. *Vaccinated Late*: *y = 0*.*0001x*^*3*^*–0*.*0111x*^*2*^ *+ 0*.*2642x + 0*.*0628*.

**Fig 2:**
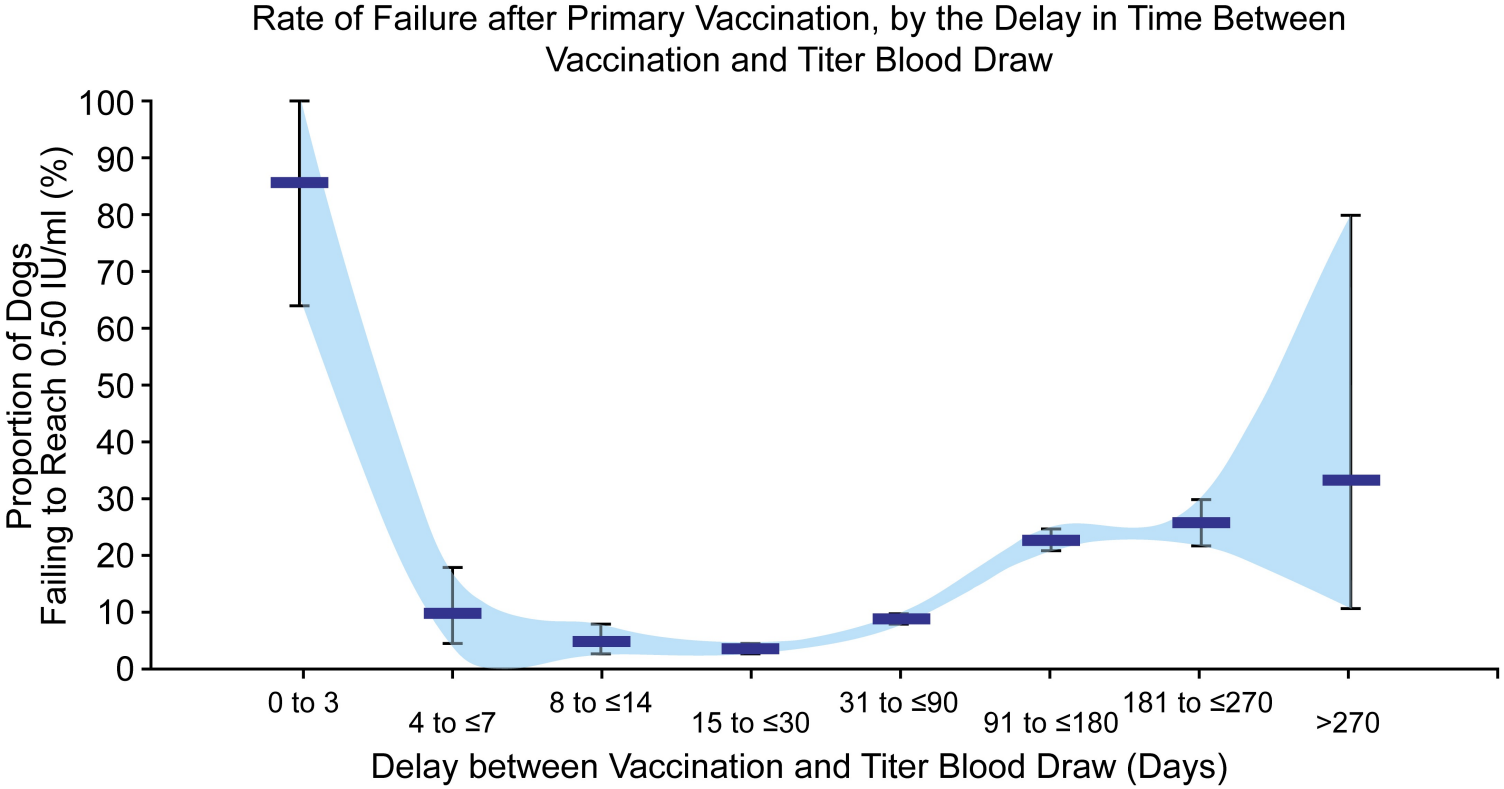
Relationship of rate of failure to delay period after primary vaccination. This figure displays the mean GMT (black bar) and associated 95% confidence intervals (wings).

Statistical analyses were performed with SAS software and OpenEpi (www.openepi.com). Variables examined included age group, time period between vaccination and blood draw, and breed size as well as pure versus mixed breed. Comparison of the frequency for the variables was analyzed using the Cochran-Mantel-Haenszel test of significance. Geometric mean titers were calculated and multivariable linear regression models were developed to identify variables that were significantly associated with individual titer levels. Serological responses were plotted by the draw interval. The serologic response was measured two ways; GMT, and proportions failing to reach 0.5 IU/ml (Figs 1 and 2, respectively). A polynomial trend line with 3 orders was determined to provide the best fit for the data. Titers for dogs which failed to achieve 0.5 IU/ml, and for which a repeat titer was conducted and captured in the database, were analyzed as described above.

## Results

The number of dogs less than 1 year of age with no documented history of vaccinations was 8,215. A total of 204 (2.5%) dogs were removed from analysis due to missing or inconsistent data in the laboratory form, for a final study sample size of 8,011 dogs (Table 1). The number of dogs that failed the FAVN test (<0.5 IU/ml) included 964 (12.0%) samples and the number of dogs that passed the FAVN test (>0.5 IU/ml) included 7,047 (88.0%) samples. A higher proportion of dogs had greater than the minimum titer required when they were vaccinated at an age greater than 16 weeks compared to dogs vaccinated early or on-time (89.6% compared to 83.4% and 84.5%, respectively) (Table 1).

**Table 1:** Rabies vaccination data- dogs less than 1 year of age given only 1 rabies vaccine.

	Samples	Percentage
Total Dogs < 1 year	13,061	100%
Total Dogs < 1 year & 1 vaccine	8,011	61.3%
Geometric Mean Titer Value	1.50 IU/ml	
Total Dogs Failed to Reach 0.5 IU/ml	964	12.0%
Total Dogs that Passed (≥0.5 IU/ml)	7,047	88.0%
Age at Primary Vaccination < 12 weeks	290	3.6%
Passed	242	83.4%*
Failed	48	16.6%*
Age at Primary Vaccination 12–16 weeks	2,238	27.9%
Passed	1,892	84.5%**
Failed	346	15.5%**
Age at Primary Vaccination > 16 weeks	5,483	68.4%
Passed	4,913	89.6%***
Failed	570	10.4%***

*Percentage out of <12 weeks group (290)

**Percentage out of 12–16 weeks group (2,238)

***Percentage out of >16 weeks group (5,483)

The geometric mean titer (GMT) for all dogs sampled was 1.50 IU/ml, irrespective of the draw interval (Table 1). Further delineation of the sample numbers into three age groups at which the primary vaccination was received is displayed in Table 2. Age at vaccination had a significant impact on antibody titer response; dogs vaccinated at >16 weeks of age had a significantly higher GMT (1.64 IU/ml, 1.57–1.72, ANOVA p < 0.01) (Table 2). There was no statistical difference in GMT between dogs vaccinated early and dogs vaccinated on-time (1.22 IU/ml compared to 1.21 IU/ml) (Table 2).

**Table 2:** Number of dog samples by titer range and age at primary vaccination.

Age at Primary Vaccination	<0.50 IU/ml	0.5–2.62 IU/ml	>2.62 IU/ml	Total Dogs	CMH* p-value	GMT	Lower 95% CL	Upper 95% CL	ANOVA p-value	Draw Interval (days, 95% CI)
< 12 weeks	48 (16.6%)	120 (41.4%)	122 (42.1%)	290		1.22	0.97	1.54		92 days (83–100)
12–16 weeks	346 (15.5%)	959 (42.9%)	933 (41.7%)	2,238		1.21	1.12	1.32		79 days (76–82)
>16 weeks	570 (10.4%)	1,965 (35.8%)	2,948 (53.8%)	5,483		1.64	1.57	1.72		64 days (62–65)
**Total**	**964**	**3,044**	**4,003**	**8,011**	**<0.01**	**1.50**			**<0.01**	

*Cochran-Mantel-Haenszel

The sex of the dog did not significantly impact failure rates for any of the three vaccination age categories (p = 0.51, 0.28, 0.12 for early, on-time, and late ages of vaccination) and was not a significant variable in the full model (Tables 3–6). Similarly, the GMT for males and females in all three age categories was non-significant, indicating that the primary immune response is not associated with sex. High responders (titers greater than 1.5 IU/ml) were more commonly seen when the titer was drawn between 8 and 30 days after vaccination, and among toy and small breed dogs (Tables 3–5).

**Table 3:** Dogs less than 12 weeks of age.

RABIES ANTIBODY TITER VALUES**							
		LOW RESPONDERS	MODERATE RESPONDERS	HIGH RESPONDERS	CMH^±^ p-value	Geometric Mean Titer	P-Value*
**Age at Vaccination: Under 12 Weeks**	**Sex**						
	Male	24 (15.9%)	63 (41.7%)	64 (42.4%)	0.51	1.15 (0.14–1.63)	*referent*
	Female	24 (17.9%)	55 (41.0%)	55 (41.0%)		1.26 (0.91–1.75)	0.80
	Unspecified	0	2 (40.0%)	3 (60.0%)		3.1 (0.98–9.58)	**0.02**
	**Time from Vaccine Administration to Titer Check**						
	≤ 3 days	0	0	0	**<0.01**	-	-
	4 to ≤ 7 days	1 (25%)	1 (25%)	2 (50%)		1.42 (0.27–7.39)	0.54
	8 to ≤ 14 days	0	0	2 (100%)		4.14 (2.89–5.93)	0.43
	15 to ≤ 30 days	3 (5.9%)	16 (31.4%)	32 (62.8%)		2.51 (1.99–3.16)	*referent*
	31 to ≤ 90 days	22 (18.3%)	50 (41.7%)	48 (40.0%)		1.21 (0.87–1.68)	**<0.01**
	91 to ≤ 180 days	19 (26.4%)	31 (43.1%)	22 (30.6%)		0.55 (0.28–1.12)	**<0.01**
	180 to ≤ 270 days	2 (5.9%)	19 (55.9%)	13 (38.2%)		2.05 (1.58–2.66)	0.12
	≥ 270 days	1 (14.3%)	3 (42.9)	3 (42.9%)		1.46 (0.67–3.19)	0.14
	**Type of Dog**						
	Mixed Breed	16 (20.3%)	23 (29.1%)	40 (50.6%)	**0.03**	1.15 (0.71–1.85)	0.66
	Pure Breed	32 (15.2%)	97 (46.0%)	82 (38.9%)		1.25 (0.96–1.64)	*referent*
	**Dog Size**						
	Toy	3 (6.5%)	21 (45.7%)	22 (47.8%)	0.46	1.99 (1.53–2.58)	0.42
	Small	9 (16.1%0	25 (44.6%)	22 (39.3%)		1.47 (0.97–2.25)	*referent*
	Medium	7 (18.9%)	18 (48.7%)	12 (32.4%)		0.95 (0.55–1.75)	0.50
	Large	11 (17.7%)	28 (45.2%)	23 (37.1%)		0.98 (0.55–1.75)	0.44
	Giant	1 (14.3%)	3 (42.9%)	3 (42.9%)		0.60 (0.03–10.70)	0.80
	Unspecified	17 (20.7%)	25 (30.5%)	40 (48.8%)		1.15 (0.72–1.82)	0.14
	***TOTAL***	***48 (16*.*6%)***	***120 (41*.*4%)***	***122 (42*.*1%)***		***1*.*22 (1*.*11–1*.*33)***	

* P-value obtained from multivariable linear regression model of all variables represented in table 3, for the prediction of the geometric mean titer (GMT):

GMT = 3.19 + female * (0.05) + sex unspecified * (1.91) + mixed breed * (0.44) + toy * (0.28) + medium * (-0.25) + large * (-0.24) + giant * (0.17) + draw delay 7 days * (-0.55) + draw delay 14 days * (0.98) + draw delay 90 days * (-0.96) + draw delay 180 days * (-1.32) + draw delay 270 days * (-0.59) + draw delay 360 days * (-1.02)

** low responders are dogs with a titer < 0.5 IU/ml, high responders are dogs with a titer >1.5 IU/ml

^±^Cochran-Mantel-Haenszel

**Table 4:** Dogs 12–16 weeks of age.

RABIES ANTIBODY TITER VALUES**							
		LOW RESPONDERS	MODERATE RESPONDERS	HIGH RESPONDERS	CMH^±^ p-value	Geometric Mean Titer	P-value*
**Age at Vaccination: 12–16 Weeks**	**Sex**						
	Male	185 (16.8%)	472 (42.8%)	445 (40.4%)	0.28	1.17 (1.04–1.32)	*referent*
	Female	159 (14.2%)	480 (42.7%)	485 (43.2%)		1.27 (1.13–1.42)	0.15
	Unspecified	2 (16.7%)	7 (58.3%)	3 (25.0%)		0.53 (0.10–2.62)	0.14
	**Time from Vaccine Administration to Titer Check**						
	≤ 3 days	7 (100.0%)	0 (0.0%)	0 (0.0%)	**<0.01**	0.01 (0.00–0.15)	**<0.01**
	4 to ≤ 7 days	1 (25.0%)	2 (50.0%)	1 (25.0%)		0.23 (0.00–35.52)	0.43
	8 to ≤ 14 days	4 (9.1%)	17 (38.6%)	23 (52.3%)		1.68 (1.00–2.81)	0.72
	15 to ≤ 30 days	28 (5.4%)	212 (40.6%)	282 (54.0%)		2.13 (1.92–2.37)	*referent*
	31 to ≤ 90 days	97 (10.9%)	384 (43.1%)	411 (46.1%)		1.56 (1.40–1.73)	**<0.01**
	91 to ≤ 180 days	131 (25.1%)	237 (45.2%)	155 (29.6%)		0.74 (0.60–0.90)	**<0.01**
	180 to ≤ 270 days	75 (31.3%)	104 (43.3%)	61 (25.4%)		0.51 (0.36–0.73)	**<0.01**
	≥ 270 days	3 (60.0%)	2 (40.0%)	0 (0.0%)		0.03 (0.00–2.44)	**<0.01**
	**Type of Dog**						
	Mixed Breed	55 (12.5%)	204 (46.5%)	180 (41.0%)	0.09	1.44 (1.25–1.65)	0.07 *referent*
	Pure Breed	291 (16.2%)	755 (42.0%)	753 (41.9%)		1.17 (1.06–1.28)	
	**Dog Size**						
	Toy	51 (12.2%)	160 (38.2%)	208 (49.6%)	**<0.01**	1.51 (1.27–1.79)	0.09
	Small	74 (14.8%)	221 (44.3%)	204 (40.9%)		1.36 (1.16–1.59)	*referent*
	Medium	43 (16.9%)	97 (38.0%)	115 (45.1%)		0.99 (0.73–1.34)	0.76
	Large	108 (20.6%)	229 (43.7%)	187 (35.7%)		0.91 (0.74–1.11)	**<0.01**
	Giant	10 (11.1%)	45 (50.0%)	35 (38.9%)		1.43 (1.01–2.04)	0.39
	Unspecified	60 (13.3%)	207 (45.9%)	184 (40.8%)		1.34 (1.15–1.56)	**0.05**
	***TOTAL***	**346 (15.5%)**	**959 (42.9%)**	**933 (41.7%)**		***1*.*21 (1*.*12–1*.*32)***	

* P-value obtained from multivariable linear regression model of all variables represented in table 4, for the prediction of the geometric mean titer (GMT):

GMT = 2.87 + female * (0.10) + sex unspecified * (-0.68) + mixed breed * (0.84) + toy * (0.18) + medium * (-0.04) + large * (-0.29) + giant * (-0.15) + draw delay 3 days * (-2.74) + draw delay 7 days * (-0.63) + draw delay 14 days * (-0.09) + draw delay 90 days * (-0.38) + draw delay 180 days * (-1.04) + draw delay 270 days * (-1.21) + draw delay 360 days * (-1.94)

** low responders are dogs with a titer < 0.5 IU/ml, high responders are dogs with a titer >1.5 IU/ml

^±^Cochran-Mantel-Haenszel

**Table 5:** Dogs greater than 16 weeks of age.

RABIES ANTIBODY TITER VALUES**							
		LOW RESPONDERS	MODERATE RESPONDERS	HIGH RESPONDERS	CMH^±^ p-value	Geometric Mean Titer	ANOVA* p-value
**Age at Vaccination: Over 16 Weeks**	**Sex**						
	Male	279 (10.0%)	1,002 (35.9%)	1,509 (54.1%)	0.12	1.69 (1.59–1.80)	*referent*
	Female	286 (10.8%)	935 (35.4%)	1,418 (53.7%)		1.60 (1.49–1.72)	0.67
	Unspecified	5 (9.3%)	28 (51.9%)	21 (38.9%)		1.44 (0.93–2.22)	0.16
	**Time from Vaccine Administration to Titer Check**						
	≤ 3 days	41 (83.7%)	4 (8.2%)	4 (8.2%)	**<0.01**	0.02 (0.01–0.06)	**<0.01**
	4 to ≤ 7 days	7 (8.4%)	27 (32.5%)	49 (59.0%)		1.65 (1.01–2.69)	0.09
	8 to ≤ 14 days	13 (4.4%)	58 (19.7%)	224 (75.9%)		2.66 (2.31–3.06)	0.16
	15 to ≤ 30 days	46 (3.1%)	379 (25.3%)	1,072 (71.6%)		2.70 (2.57–2.85)	*referent*
	31 to ≤ 90 days	160 (7.4%)	821 (37.9%)	1,188 (54.8%)		1.86 (1.75–1.99)	**<0.01**
	91 to ≤ 180 days	231 (21.4%)	523 (48.4%)	327 (30.3%)		0.86 (0.75–0.98)	**<0.01**
	180 to ≤ 270 days	72 (23.3%)	153 (49.5%)	84 (27.2%)		0.75 (0.59–0.97)	**<0.01**
	≥ 270 days	0	0	0		-	**-**
	**Type of Dog**						
	Mixed Breed	94 (8.3%)	414 (36.4%)	628 (55.3%)	**0.03**	1.80 (1.64–1.99)	0.59
	Pure Breed	476 (11.0%)	1,551 (35.7%)	2,320 (53.4%)		1.61 (1.52–1.69)	*referent*
	**Dog Size**						
	Toy	121 (9.2%)	452 (34.4%)	740 (56.4%)	**<0.01**	1.74 (1.58–1.92)	**<0.01**
	Small	137 (10.7%)	452 (35.4%)	687 (53.8%)		1.68 (1.53–1.83)	*referent*
	Medium	55 (10.2%)	196 (36.5%)	286 (53.3%)		1.71 (1.48–1.96)	0.98
	Large	132 (12.8%)	383 (37.2%)	514 (50.0%)		1.40 (1.24–1.57)	**0.04**
	Giant	29 (16.8%)	61 (35.3%)	83 (48.0%)		1.20 (0.87–1.66)	0.32
	Unspecified	96 (8.3%)	421 (36.5%)	638 (55.2%)		1.80 (1.64–1.98)	0.81
	***TOTAL***	**570 (10.4%)**	**1,965 (35.8%)**	**2,948 (53.8%)**		***1*.*64 (1*.*57–1*.*72)***	

* P-value obtained from multivariable linear regression model of all variables represented in table 5, for the prediction of the geometric mean titer (GMT):

GMT = 3.27 + female * (0.02) + sex unspecified * (-0.30) + mixed breed * (0.19) + toy * (0.18) + medium * (-0.002) + large * (-0.14) + giant * (-0.13) + draw delay 3 days * (-2.68) + draw delay 7 days * (-0.30) + draw delay 14 days * (0.14) + draw delay 90 days * (-0.55) + draw delay 180 days * (-1.42) + draw delay 270 days * (-1.54)

** low responders are dogs with a titer < 0.5 IU/ml, high responders are dogs with a titer >1.5 IU/ml

^±^Cochran-Mantel-Haenszel

**Table 6:** Multivariable linear regression model for the prediction of geometric mean titer after primary rabies vaccination.

Parameter	Parameter Estimate	t Value	Pr > t
**Time from Vaccine Administration to Titer Check**			
≤ 3 days	**-2.66**	**-12.48**	**<0.01**
4 to ≤ 7 days	-0.29	-1.69	0.09
8 to ≤ 14 days	0.13	1.42	0.15
15 to ≤ 30 days	*referent group*		
31 to ≤ 90 days	**-0.52**	**-11.74**	**<0.01**
91 to ≤ 180 days	**-1.31**	**-25.3**	**<0.01**
180 to ≤ 270 days	**-1.39**	**-18.7**	**<0.01**
≥ 270 days	**-1.41**	**-3.08**	**<0.01**
**Timing of Vaccine Administration**			
Early	0.13	1.36	0.17
On Time	*referent group*		
Late	**0.26**	**6.57**	**<0.01**
**Sex**			
Male	*Variable removed due to lack of significance in model*		
Female			
Unspecified			
**Type of Dog**			
Mixed Breed	*Variable removed due to lack of significance in model*		
Pure Breed			
**Dog Size**			
Toy	**0.18**	**3.36**	**<0.01**
Small	*referent group*		
Medium	-0.02	-0.33	0.74
Large	**-0.19**	**-3.47**	**<0.01**
Giant	-0.15	-1.43	0.15
Unspecified	0.05	0.86	0.39
**Intercept**			
Intercept	**3.00**	**53.54**	**<0.01**

Model degrees of freedom: 14

F Value 79.00 (p<0.01)

R-square: 0.12

Mixed breed dogs with primary vaccination on time and late were more likely to pass the FAVN compared to pure breed dogs (12.5% vs 16.2% [p = 0.09] and 8.3% vs 11.0% [p = 0.03], respectively). Mean GMTs were higher for mixed breed dogs when vaccinated on-time or late, however this difference was not significant at the level of p<0.05. These associations with mixed breed dogs were not observed when dogs were vaccinated too early.

Among dogs vaccinated earlier than 12 weeks of age, dogs size was not significantly associated with FAVN failure (p = 0.46) (Table 3). Both failure rates and GMTs were significantly associated with breed size when dogs were vaccinated on-time or late (p < 0.01 for both measures) (Tables 4 and 5). Generally, toy and small breed dogs had a higher probability of a passing FAVN and higher GMT when compared to larger dog breeds.

The draw interval was significantly associated with failure rates for all three vaccination age categories (Tables 3–5). The GMT showed an inverse parabolic association, with values lowest for short draw intervals, highest for moderate draw intervals, and declining for long draw intervals. Regardless of age at vaccination, failure rates were lowest when the draw interval was between 8 and 30 days (failure rate range 3.1%–9.1%). Failure rates were highest when the draw interval was 3 days or earlier (range 87–100% failure rate) and draw intervals greater than 90 days (range 5.9%–60% failure rate). The majority (75–91.6%, depending on age at vaccination) of dogs had a titer greater than 0.5 IU/ml by 4–7 days after primary vaccination.

A multivariable linear regression model for estimating the GMT was developed and identified 3 significant variables: draw interval, age at vaccination, and breed size. The dog’s sex and mixed-breed status were not significant in this full model and were removed. When compared to draw intervals of 14–30 days, the GMT significantly decreased (p < 0.05) when the interval was less than 4 days or greater than 30 days. On-time and early vaccination had no significant impact on GMT, however GMT is significantly increased with late vaccination (p < 0.01). When compared to small breed dogs, GMT was significantly higher for toy dogs and significantly lower compared to large breed dogs (p < 0.01 for both).

The relationship between primary vaccination and antibody titer was best fit by a 3-tier polynomial model. Two models had generally good fit when considering that only draw-interval was considered in the regression analysis: on-time, and late (r^2^ = 0.50, and 0.77, respectively) (Fig 1). For animals with primary vaccination after 12 weeks of age, the polynomial model predicted that an adequate FAVN titer would be reached around day 3 post-vaccination. For all three vaccination age categories, the peak titer was expected to occur around 12–18 days post-vaccination, and drop below 0.5 IU/ml at 160 days after vaccination.

When failure rate was analyzed without regard to age at vaccination, the rate of failure to reach 0.5 IU/ml was greatest when titers were drawn within three days of primary vaccination (85.7%, 95% CI 64.0%–100%) (Fig 2). Failure rate was lowest when blood was drawn 15–30 days after vaccination (3.7%, 95% CI 2.9%–4.6%). Larger confidence intervals around the failure rate were noted for very short and very long draw intervals.

**Fig 3:**
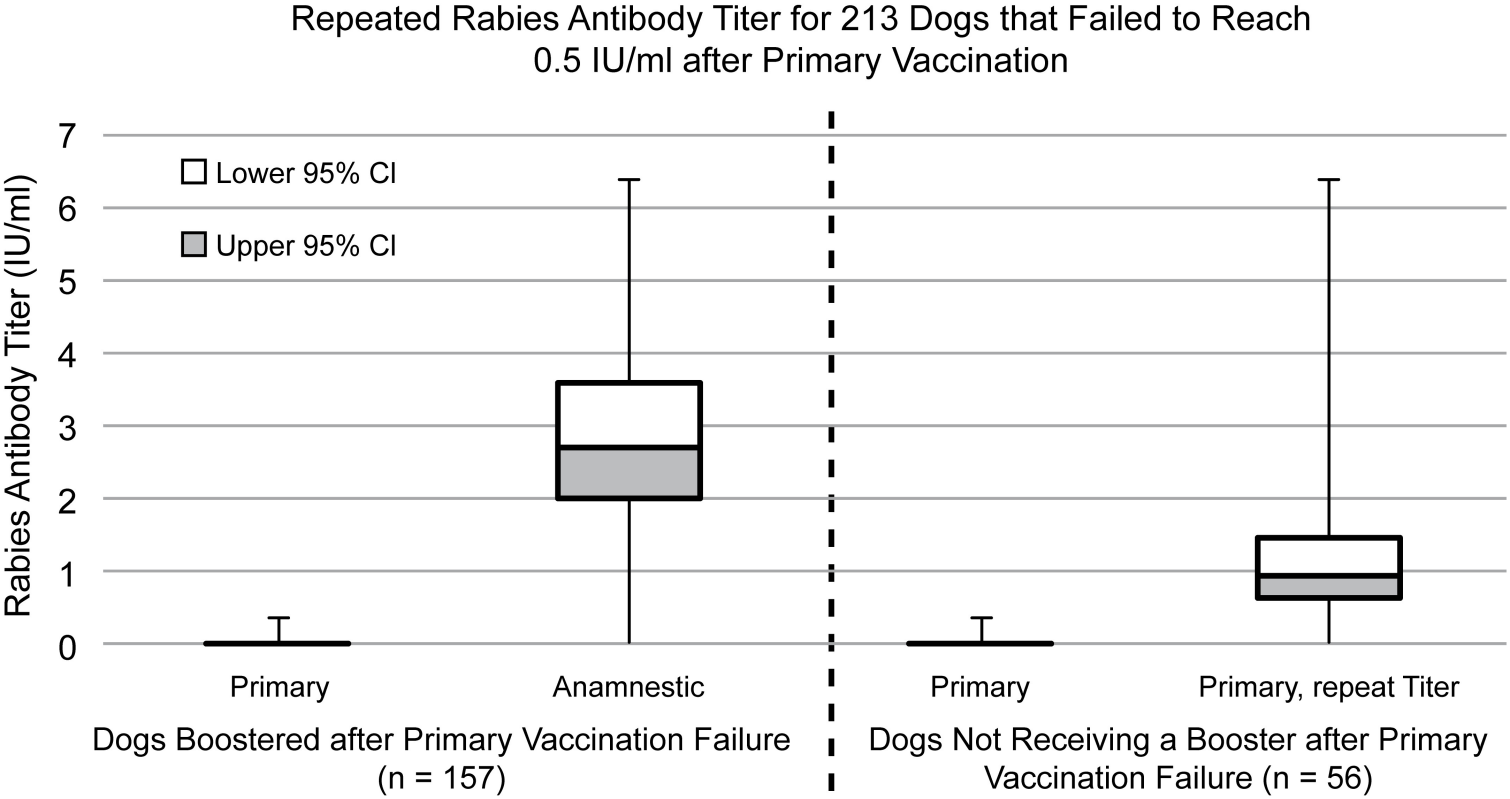
Comparison of rabies antibody titers for dogs boosted versus not boosted after primary vaccination failure.

Among dogs that failed to reach 0.5 IU/ml upon initial FAVN, and for which a repeat titer was recorded (n = 213), 195 subsequently exceeded this titer level (91.5%) (Fig 3). Of these 213 failures, 157 received a booster vaccine and 56 did not. Upon repeat titer measurement, animals that received a booster vaccine after failure had a GMT of 2.7 IU/ml (95% CI 2.07–3.57), compared to a GMT of 0.97 IU/ml for dogs that did not receive the booster (95% CI 0.64–1.48). Boosted dogs passed upon repeat titer testing 96.8% of the time. Dogs that did not receive a booster passed 76.8% of the time.

## Discussion

A dog’s ability to neutralize rabies virus after vaccination is a reliable indicator of a dog’s risk of developing rabies if exposed. Ensuring that adequate serologic responses have occurred after vaccination is important both in rabies endemic countries (influences vaccination program coverage) as well as in rabies-free countries (provides evidence that an animal entering the rabies-free zone will not harbor the virus). Findings from this study are based on analyses of data collected over a five year period from the KSU rabies diagnostic laboratory. The KSU rabies diagnostic laboratory is one of the highest-volume rabies serology facilities in the world. As such, this analysis is one of the largest systematic evaluations of the serologic response in dogs after primary rabies vaccination. The standardized data collection process at KSU enabled evaluation of several key factors that can influence the immune response to primary vaccination. The findings from this study confirm some previously published conclusions regarding failure rates in rabies vaccine naïve animals, but also provides new evidence for important considerations when determining when vaccines should be administered and titers checked.

The overall threshold-titer failure rate for the dogs analyzed in this study (12.0%) was consistent with one other published study of primary vaccination (14%) [10]. However, variations in failure rates have been reported, with some dog populations failing as low as 4% of the time [11]. Although we were not able to collect data on vaccine type administered for each subject for this study, variations in the type of vaccine used, recommendations for timing of vaccination, and the genetics of the dog population are likely to influence inter-study failure rates.

Several biological processes occur shortly after birth and through the first few months of development that can have an impact on an animal’s ability to respond to antigen stimulation. Shortly after birth, animal immune systems are still in a nascent, developmental phase, and they may have circulating maternal antibodies [15,16]. The finding that dogs vaccinated before 16 weeks of age had a lower rate of passing the FAVN and a lower GMT is likely due to a combination of factors: immune system development, presence of maternal antibody, and overall health development. A better understanding of these biological interactions may help improve vaccination timing. However, given the low overall rate of failure among all age groups and high probability of a passing titer upon booster, this may be an academic pursuit that is unlikely to impact public health recommendations.

While titer outcomes were better when vaccination occurred after 16 weeks of age, there was no statistical difference in failure rate nor the GMT when animals were vaccinated on-time or earlier than 12 weeks. This finding supports recent World Health Organization recommendations that mass rabies vaccination campaigns should vaccinate all dogs that present to a vaccination team, regardless of age [1]. In the United States most rabies vaccines are licensed for dogs older than 12 weeks of age, and most regulations require dogs to be vaccinated by 16 weeks of age. In the United States and other rabies-free countries, policy makers should balance the risk of rabies exposure to dogs to the expected immune response at the age of vaccination. Delaying the required vaccination age beyond 16 weeks increases the time in which that dog is susceptible to rabies while only improving the failure rate by 5%. Given the grave consequences of rabies infection, current licensure and vaccination recommendations appear adequate for countries with rabies epidemiology similar to the United States [17].

Mixed breed dogs had an improved titer response and failure rate when compared to pure breed dogs in this study (Tables 3 and 4). There are reports that mixed breed dogs are genetically more robust and less prone to inherited disorders and disease [18,19]. There are likely exceptions to this simplistic generalization as some breeds were developed through rapid inbreeding and the genetic ‘health’ of those dogs may be questionable. Conversely, some pure breeds (Anatolian) were bred over thousands of years, and are not predisposed to as many genetic disorders. Since antigenic recognition and antibody production are tied to genetics, this is an association that should be considered. Further distinction of breed influence on immunologic response to rabies vaccination may help identify specific breed or breed-categories that are at higher risk of titer failure.

Smaller dog size was associated with more favorable titer outcomes. As mentioned earlier, specific breed genetics may influence this finding. Another likely factor in this reported observation is the role of dose-response to the rabies vaccine. All dogs, regardless of size, receive the same dose of rabies vaccine (1 ml per US licensed products). The concentration of antigen can vary between products and batches [20]. However, assuming no bias in vaccine products in the study population and an antigenic load of 1 IU per vaccine, the dosage of antigen dogs receive varies greatly between toy breeds and giant breeds; a 1.5 kg Chihuahua puppy receives a rabies dosage of 0.7 IU/kg, while a 25 kg Saint Bernard puppy receives a rabies dosage of 0.04 IU//kg. This dose response effect has been noted in other publications [11]. In humans the dose response was clearly demonstrated by administration of serial dilutions of rabies vaccine in a study by Berens et al., supporting the minimum antigen dosage for human rabies vaccine [21]. Use of the minimum dose designated in vaccine approval studies is clearly required. Providing a larger antigenic load or 2 site vaccination for larger breed dogs may help decrease the failure rate after primary vaccination.

The most significant factor related to having an adequate titer was the time between vaccination and when blood was drawn. Among the population of dogs in this study, the probability of a passing titer was greatest when taken between 8 and 30 days after vaccination. It is notable, that though it is commonly thought only an anamnestic response is characterized by a rapid, robust immune response, a large proportion of rabies vaccine-naïve dogs demonstrated just the same rapid rise in titer within seven days; 61% with a documented serological response above 0.5 IU/ml. The majority of dogs failed to reach an adequate titer within the first 3 days of primary vaccination, and failure rates increased if the draw interval was greater than 90 days. These findings are consistent with other published literature, and reinforce the ideal timing that should be considered for blood draws when they are taken for documentation of a titer >0.5 IU/ml.

When a dog fails a titer check, owners have two options: 1) booster the dog and re-check the titer *or* 2) wait for the immune system to produce more antibody and re-check the titer at a later date. Booster vaccination comes at an added expense and exposes the dog to potentially unnecessary vaccine biologics that hold a small risk of adverse reaction. This study shows that three-quarters of dogs will pass a titer response simply by increasing the draw interval, in the absence of a booster dose. However, the highest probability of achieving an adequate immune response after a failure is a booster vaccine dose. Owners should consider the cost of repeat vaccination, time constraints, and potential (low risk) for an adverse reaction when deciding how to respond to a dog that has failed primary vaccination. This study provides some quantitative values for which these decisions can be based.

In today’s interconnected world, people and their pets can now traverse continents in a matter of hours. While this progress has eased international trade and communications, a pathway for infectious diseases to travel rapidly across vast distances has emerged. Countries that have undergone the financial and physical responsibility to eliminate canine rabies virus have financial and health incentives to protect that status. Thus, most canine rabies-free countries require evidence of vaccination for dogs entering their country. Findings here support previous studies, with a larger dataset, that the majority of U.S. dogs will respond favorably to primary vaccination and the appropriate time to draw a titer is 8–30 days after vaccination. A dose-response effect was noted in this analysis, and may suggest that higher doses of vaccine may be necessary for larger breed dogs to decrease the risk of vaccine failure. In the event of a failure, most dogs will achieve an adequate serologic response if the draw interval is extended (even in the absence of booster vaccination). However, booster vaccination after failure is the best way to ensure an animal passes a repeat titer test. Several variables analyzed in this study show strong correlation with the GMT and failure rates; development of predictive tools for veterinarians may help tailor vaccination and titer recommendations to help improve failure rates and reduce costs for dog owners.

## Supporting information

S1 Dataset A listing of all dog serum samples included in the analysis described in the paper with the following associated information per sample. Case number, name, breed abbreviation (breed1), breed (breed2), unique ID, Blood draw date (DRAWdate), gender (M = male, F = female), id, date of birth (DOB), rabies vaccination date (vax1date), dog size, FAN result in IU/mL) (TITER), PASS/FAIL (1 = Pass, ≥0.5 IU/mL; 0 = Fail, <0.5 IU/mL), consecutive number order of the sample in the dataset (#order original).
(XLSX)
